# High-resolution spatial transcriptomics of adult and pediatric human liver with Visium HD

**DOI:** 10.1038/s41597-026-07597-2

**Published:** 2026-06-24

**Authors:** Faizan Hasan, Rachel D. Edgar, Jawairia Atif, Diana Nakib, Cornelia Thoeni, Amanda Ricciuto, Blayne A. Sayed, Ian McGilvray, Gary D. Bader, Sonya A. MacParland

**Affiliations:** 1 Ajmera Transplant Centre, Toronto General Research Institute, University Health Network, Toronto, Ontario Canada; 2 Department of Medical Biophysics, University of Toronto, Toronto, Ontario Canada; 3 Department of Molecular Genetics, University of Toronto, Toronto, Ontario Canada; 4 Donnelly Centre, University of Toronto, Toronto, Ontario Canada; 5 Department of Immunology, University of Toronto, Toronto, Ontario Canada; 6 Department of Pathology and Lab Medicine, Nova Scotia Health Authority, Halifax, Nova Scotia Canada; 7 Dalhousie University, Faculty of Medicine, Halifax, Nova Scotia Canada; 8 Division of Gastroenterology, Hepatology & Nutrition, Hospital for Sick Children, University of Toronto, Toronto, Ontario Canada; 9 Princess Margaret Cancer Centre, University Health Network, Toronto, Ontario Canada; 10 Department of Computer Science, University of Toronto, Toronto, Ontario Canada; 11 Lunenfeld-Tanenbaum Research Institute, Sinai Health System, Toronto, Ontario Canada; 12 CIFAR Multiscale Human Program, CIFAR, Toronto, Ontario Canada; 13 Department of Laboratory Medicine and Pathobiology, University of Toronto, Toronto, Ontario Canada

## Abstract

The liver is composed of diverse cell populations that coordinate essential metabolic and immune functions. Single-cell transcriptomics has advanced characterization of liver cellular composition, but dissociation of tissue to single-cells can introduce biases through the enrichment or depletion of cell types. Spatial transcriptomics is a complementary approach to avoid inherent bias for cell populations and to add important spatial context. The Visium HD spatial transcriptomics technology from 10X Genomics enables high-resolution spatial mapping of gene expression in tissue samples with a bin width of 2 µm enabling quantification of transcripts at a sub-cellular resolution. We applied Visium HD to three healthy human liver donor samples, from two adult and one pediatric donor. We identified cell types by clustering 8 µm bins and integration with single-cell reference maps. Differential expression analyses identified spatially distinct gene expression resulting in development of a high-resolution map of the liver. This resource provides cell-level and spatially-resolved insights into the cellular and anatomical heterogeneity of the liver to serve as a resource for researchers to identify disease-specific spatial signatures and novel therapeutic targets.

## Background & Summary

The liver plays a critical role in metabolism and immune function. These crucial roles are diminished in chronic liver diseases, leading to over two million deaths annually worldwide due to liver failure^1^. Single-cell transcriptomics has provided insights into the cellular composition of livers in health and disease, but is biased due to cell type-specific enrichment or depletion during the single-cell dissociation process. Previous work has highlighted difficulties in capturing specific populations such as cholangiocytes and hepatocytes^2^. In the cells captured, the process of dissociation has been shown to impact gene transcription^3^.

Spatial transcriptomics is a promising approach that does not have inherent bias for cell populations, and adds important spatial context. Spatial transcriptomics has been shown to capture important patterns of zonation in the human liver^4^, a dynamic feature important for understanding basic liver functions and alterations in liver disease processes. Until recently, spatial transcriptomic technologies have been low-resolution (Visium, 55 µm) potentially mixing signals from multiple cells and multiple cell types. The latest spatial transcriptomic technology from 10X Genomics, Visium HD, enables high-resolution (2 µm) spatial mapping of gene expression in tissue samples, offering a sophisticated platform for exploring the cellular composition of the liver.

Here we assayed samples from three neurologically deceased donor livers, with no evidence of histopathological liver disease. This data is at higher resolution than previous whole-transcriptome spatial datasets of healthy human liver^5^. The increased resolution, achieved using the probe-based Visium HD platform rather than whole-transcriptome capture, enables the identification of rare and transcriptionally distinct cell populations within the liver zonation structure. While we do see variation in data quality across the three samples, we provide these data as a resource for better understanding of the cellular and spatial liver heterogeneity.

## Methods

**Fig. 1:**
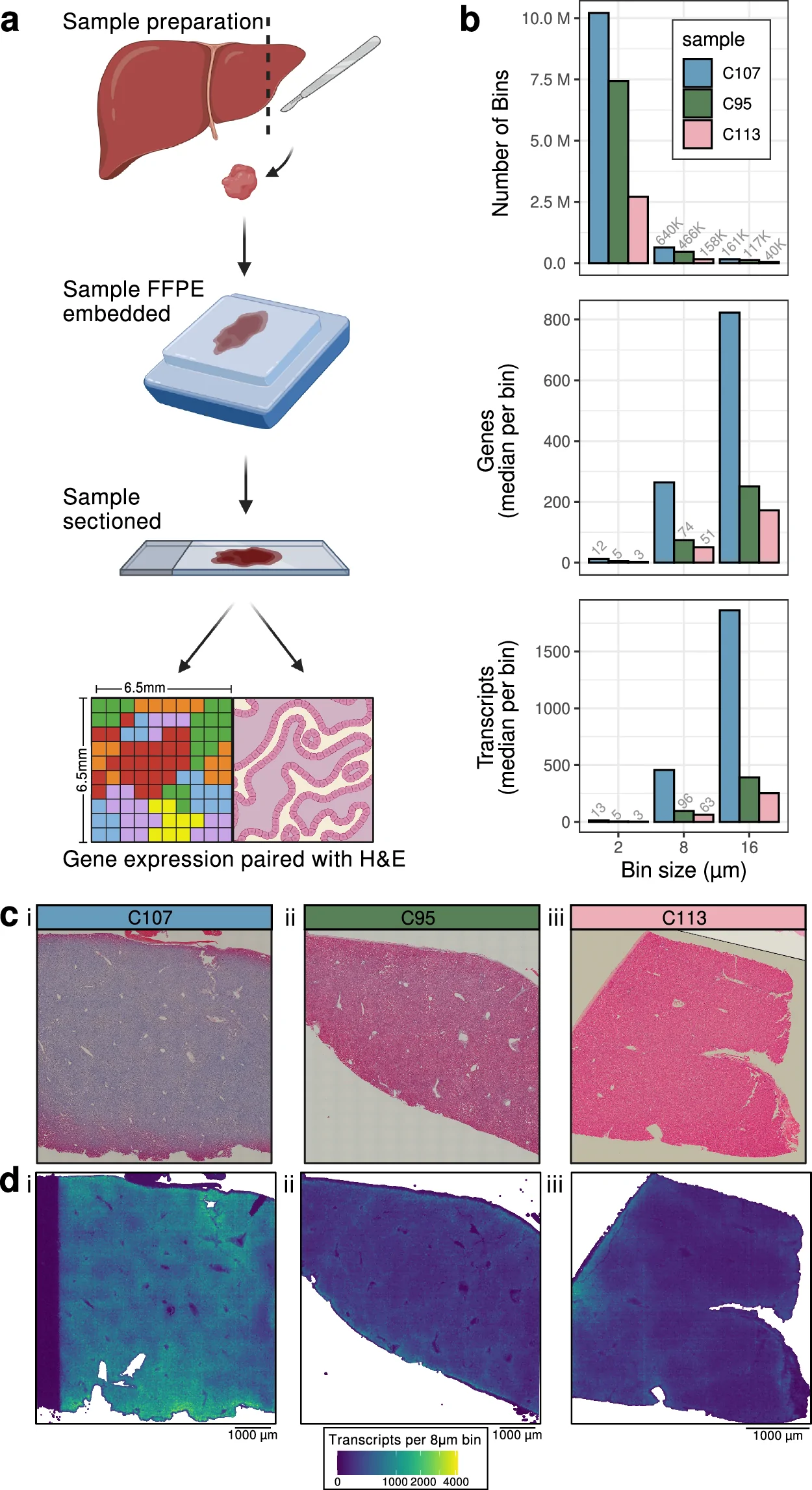
Sample processing of human liver for Visium HD resulted in three samples of varying quality. (**a**) Sample processing workflow. (**b**) Quality metrics of three samples at three bin sizes. Values are labeled above bars that are too short to be read directly from the y-axis. (**c**) H&E staining of samples processed with Visium HD. (**d**) Location of 8 µm bins coloured by number of UMIs.

Figure 1a provides an overview of the liver spatial transcriptomics data generation which involves the collection of human liver tissue, preparing tissue sections, and Visium HD sample processing.

### Human liver sample collection

Healthy human liver tissue from the caudate or right liver lobe was obtained from neurologically deceased donor livers (Table 1). Donated livers had no evidence of histopathological liver disease supported by the hematoxylin and eosin (H&E) staining of the samples^6^. However, liver tissue from neurologically deceased donors may exhibit transcriptional changes related to hemodynamic instability, inflammatory activation, or damage from ischemia events^7^. These factors represent a potential limitation when interpreting transcriptional patterns in otherwise healthy liver tissue.

**Table 1:** Spatial transcriptomic sample information.

Sample	Age	Sex	Tissue	Storage	Reads	Sequencing saturation	Spacer Ranger version	Associated data previously published
C107	51	M	Caudate	Stored for 1 year at 4 °C	929,681,741	58.9%	3.0.0	None
C95	47	F	Caudate	Stored 2 years; 1 year room temperature 1 year 4 °C	333,465,658	81.0%	3.0.0	Xenium spatial^17^
C113	15–19	M	Right lobe	Stored for 2 years at 4 °C	225,446,253	93.1%	3.0.1	Dissociated single-cell^17^

Fresh human liver samples were collected at the Hospital for Sick Children and Toronto General Hospital transplant centres. Liver tissue was prepared for Visium HD spatial transcriptomics by first processing into formalin-fixed, paraffin-embedded (FFPE) blocks which were then sectioned into 5 µm thick slices and mounted onto 6.5 mm by 6.5 mm Visium HD slides (Visium HD v1 - FFPE; probe set v2.0) following the Visium HD Gene Expression User Guide.

Samples were sequenced on an Illumina NovaSeq X system. The Visium HD spatial transcriptomic data were sequenced to the depths between 300–950 million reads (Table 1). As sample C107 was initially only sequenced to a saturation of 34.4% the library was sequenced again for a final saturation of 58.9%. Reads were mapped to the human transcriptome (GRCh38-2020-A) and expression was quantified using Space Ranger software (Table 1). One sample (C113) was part of a tissue microarray (TMA) with three diseased liver samples included in the Visium HD capture area. Those diseased samples are not included in the sequencing data presented here, but are present in image files to enable future re-processing of the samples if needed.

### Ethics statement

Healthy adult samples were collected with institutional ethics approval from the University Health Network (REB# 14-7425-AE) and the healthy pediatric sample through the Hospital for Sick Children (REB# 1000064039). This research was conducted in accordance with the Declarations of Helsinki and Istanbul, with written informed consent obtained from participants or, for pediatric or deceased donors, from their legal guardians or representatives. Participants or their guardians/representatives consented to the collection, generation, and sharing of genomic data for research purposes. Consent procedures addressed potential privacy risks associated with genomic data sharing. The approved ethics protocols allowed for dissemination of de-identified data under an open license.

### Annotation of cell type in 8 µm bins

Of the three samples processed, C107 had markedly higher quality, with twice as many genes and transcripts captured per bin (Fig. 1b–d). With only a few samples, it is uncertain why there was such variability in quality, but some sample storage information is available in Table 1.

**Fig. 2:**
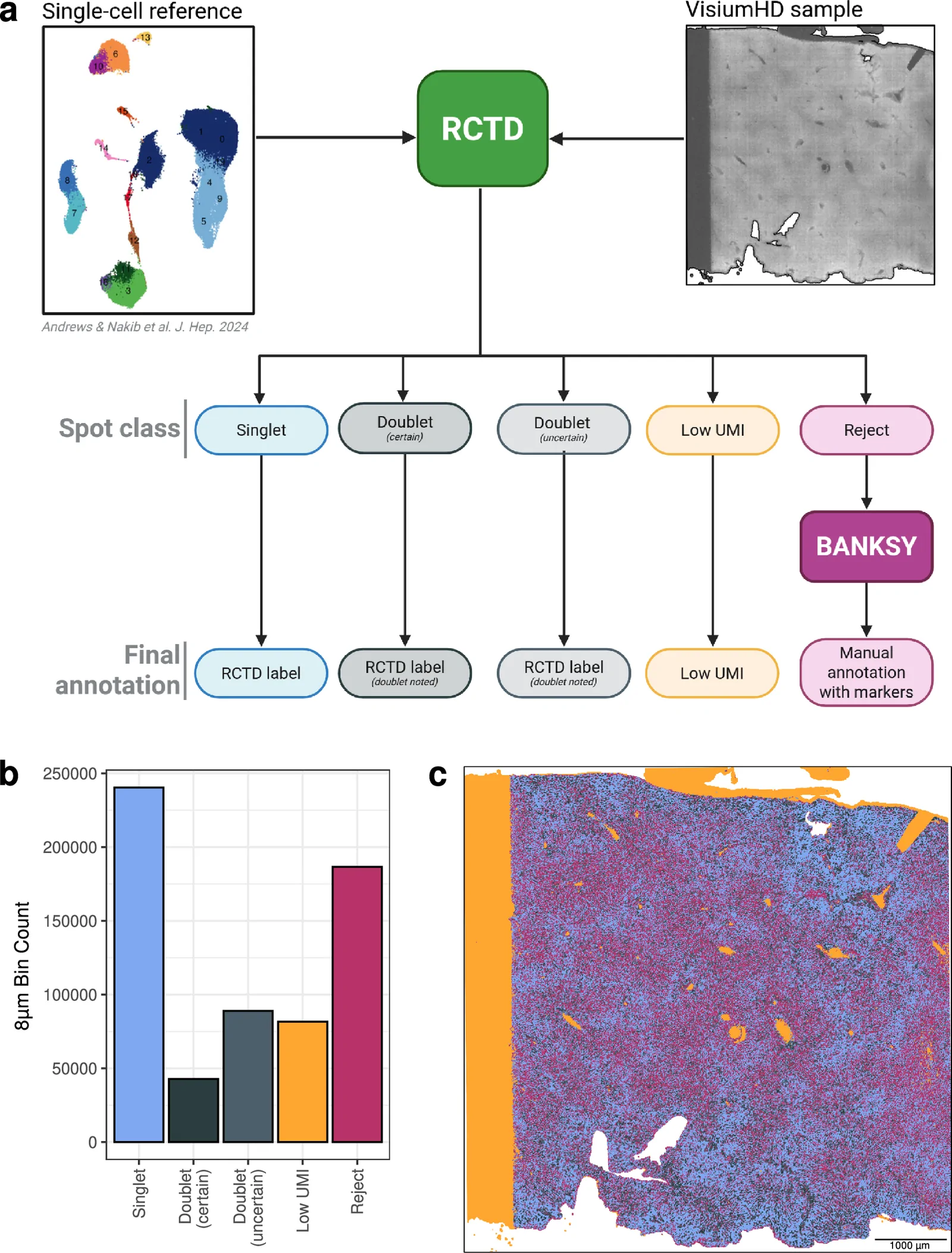
Annotation process for 8 µm bins of sample C107. (**a**) Workflow for cell annotation based on RCTD and BANKSY clustering when bins were rejected by RCTD. (**b**) Spot class output of RCTD. (**c**) Location of 8 µm bins coloured by RCTD spot class.

**Fig. 3:**
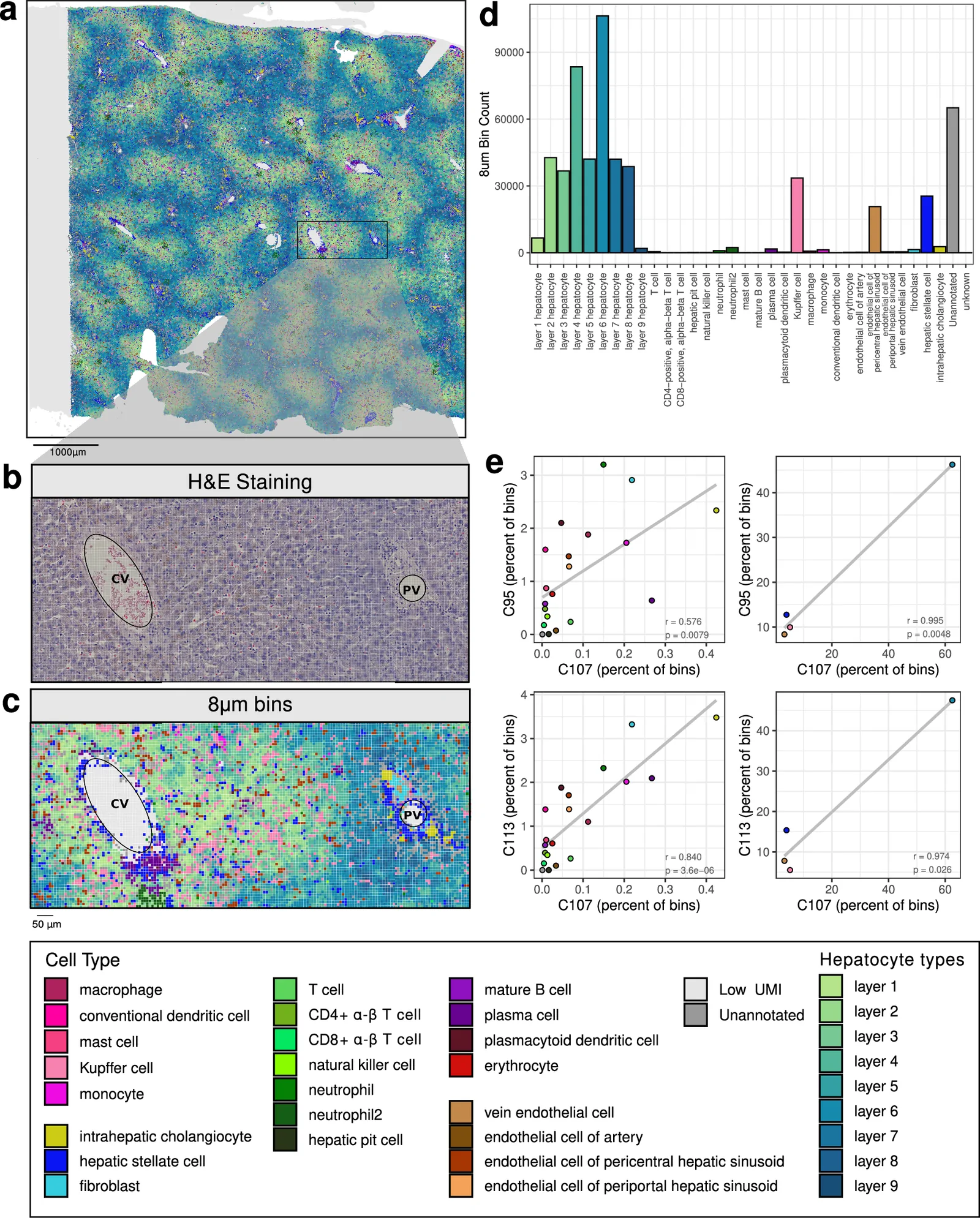
Expected cell types of the human liver can be captured, in their expected proportions and locations, using Visium HD. (**a**) Cell type annotation of the 8 µm bins. (**b**) Zoom-in of a representative central vein (CV) to portal vein (PV) axis. (**c**) Associated H&E staining of the same area. (**d**) Counts of 8 µm bins across the annotated cell types. (**e**) Correlation of cell type proportions between samples C107 and C95 (top) or C113 (bottom). Cell types are stratified based on whether they comprise more than 3% of total bins (right panels) to allow appropriate scaling. Pearson correlation coefficients and associated p-values are shown for each panel.

**Fig. 4:**
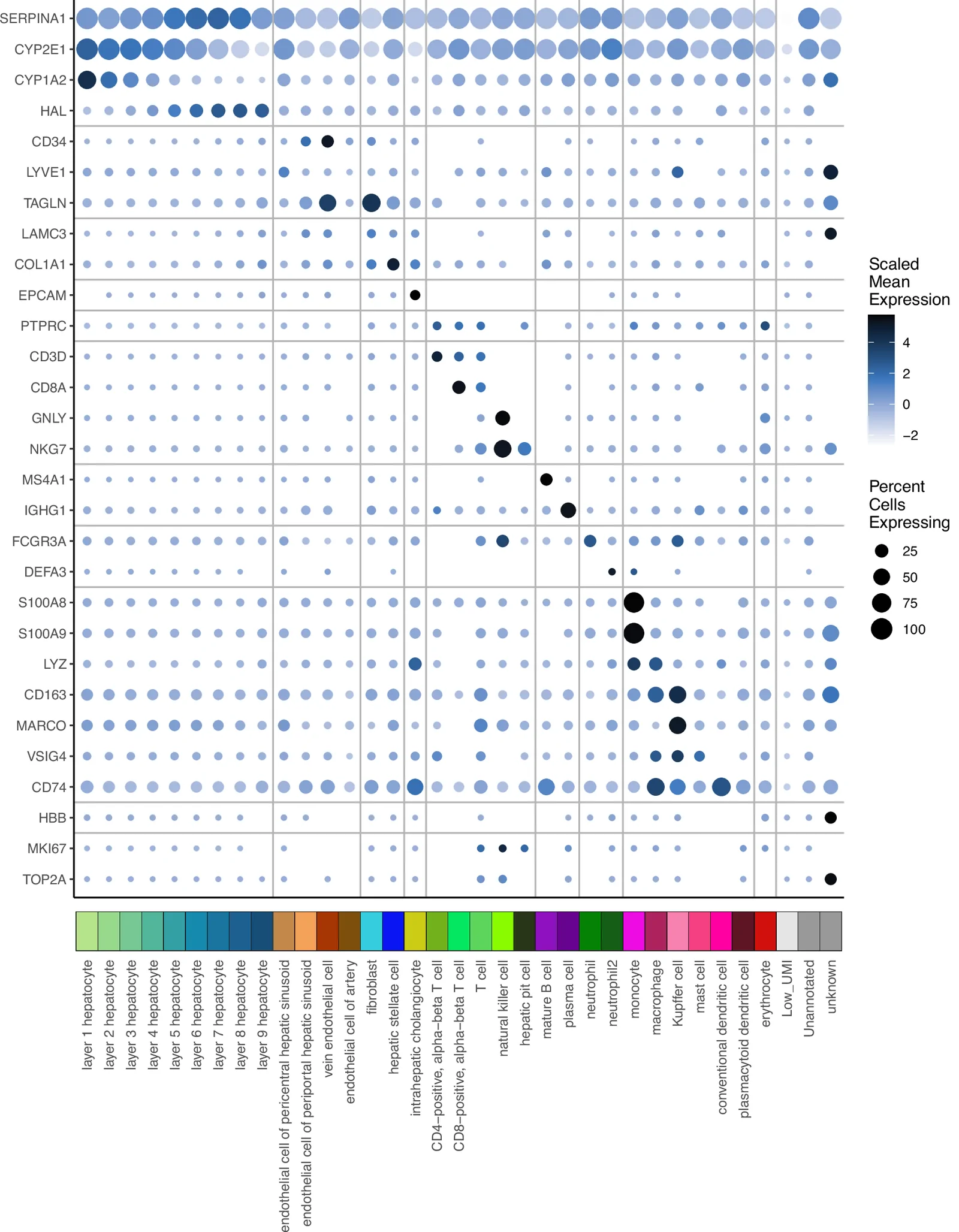
Expression of markers validates the cell type labels from the cell annotation pipeline. Gene expression of key markers used for cell type annotation. The colour of the points represents the gene expression level and size represents percent of cells of a type expressing the gene at all.

To annotate bins with a cell type, an existing liver single-cell RNA-seq reference was mapped onto the spatial data (Fig. 2a). The published human liver single-cell reference used included 89,637 cells, with 27 annotated cell types^4^. The Robust Cell Type Decomposition (RCTD)^8^ software was used to estimate cell type mixtures in spatial spots. To limit technical artefacts, low-quality bins with fewer than 100 UMIs were labelled as such in the bin annotation and filtered out from subsequent analysis (this removed 13% of all bins for C107 which were mainly around the edges and in the lumen Fig. 1d,i) before cell type annotation of bins (Fig. 2b). Spot class labels were assigned to bins using RCTD. The RCTD spot class “*singlet*” refers to bins predicted to contain only one cell type, whereas “*doublet-certain*” and “*doublet-uncertain*” refer to bins predicted to contain two cell types. Out of the high-quality bins, 67% were assigned an RCTD spot class and a cell type annotation (Figs. 2b,c, 3a–d). As noted, samples C95 and C113 were of lower quality than C107, with only 47.2% and 8.9% of their 8 µm bins, respectively, passing the RCTD quality threshold of a minimum of 100 UMIs. Despite this reduced coverage, the proportions of cell types in the high-quality bins were significantly correlated between C107 and the other two samples, suggesting biological signals are still present in these samples (Fig. 3e). For the 33% of bins not well mapped to a cell type in the single-cell reference (i.e. RCTD “*rejects*”), these bins were clustered spatially using BANKSY^9^ spatially aware clustering followed by manual annotation of distinct cell types using known cell type markers and spatial context (Figs. 3, 4). Most RCTD “*rejects*” (63%) could be annotated as hepatocytes, using the BANKSY clusters and known markers. Although correlated biological signals were observed across samples, detailed downstream analyses were focused on sample C107 due to its substantially higher data quality.

Liver tissue has defined spatial structure^10^ with hepatocytes arranged along a portal-to-central axis that generates metabolic and transcriptional gradients, a phenomenon known as liver zonation. We constructed a liver zonation axis in this data by dividing all bins into nine zonation layers, using a previously described method^11^. In brief, hepatocyte zonation scores were calculated using established marker genes^11^ and the *AddModuleScore* function from Seurat^12–14^. Bins were stratified into nine layers based on these scores. Based on these zonation scores individual bins were split into nine zonation layers. This zonation annotation was then smoothed by reassigning the zonation layer for a bin to the median zonation layer of its neighboring bins, as previously described^11^. Layer 1 corresponds to the most pericentral regions (near the central vein), layer 8 to the most periportal regions (near the portal triad), and layer 9 to regions more dense in fibroblasts and mainly but not exclusively periportal. For hepatocytes, the zonation layer was appended to the cell type name in the final annotation. Zonation of all genes across these layers was measured as differential expression between layers using the *FindAllMarkers* function from Seurat^12–14^ (with the default parameters except setting *logfc.threshold* = 0 and *min.pct* = 0). Zonated genes were identified using a Wilcoxon rank-sum test, with significance defined as a Bonferroni-adjusted p-value < 0.005 and an absolute log2 fold change >1.

**Fig. 5:**
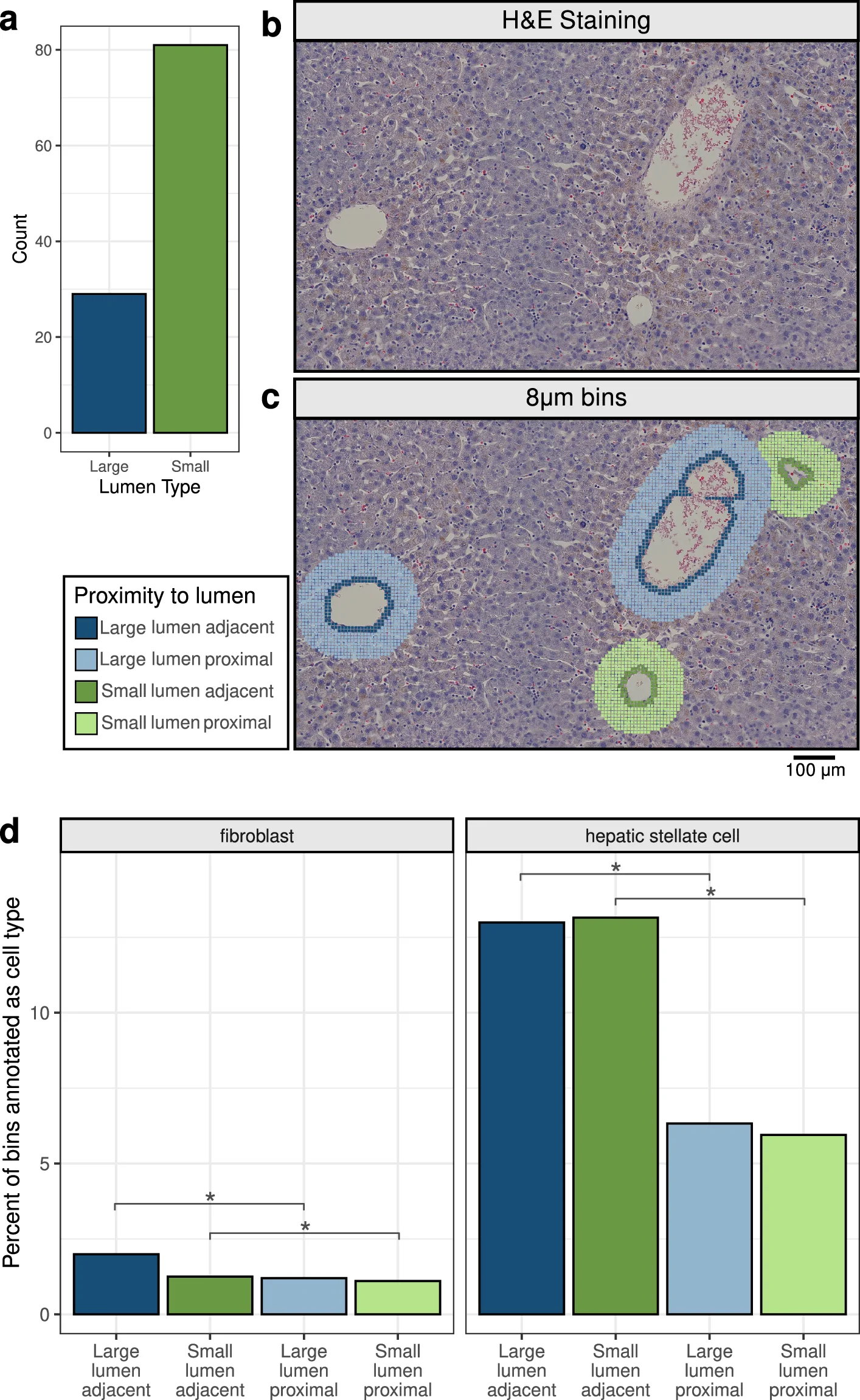
Lumen size can be quantified and associated with cell type composition. (**a**) Distribution of lumen sizes among 110 lumens identified in the tissue. (**b**) H&E staining of a representative region containing several lumens. (**c**) Association of bins by proximity to lumen of two sizes at the 8 µm bin resolution. (**d**) Percent of fibroblasts and hepatic stellate cells in regions adjacent (≤16 µm) or proximal (≤80 µm) to lumen across the entire tissue. *p < 0.05; two-way ANOVA.

Pairing Visium HD transcriptional information and H&E images enabled the examination of structural features in the human liver. Lumen regions were identified in the H&E image based on colour intensity. The space in the image not covered by tissue (around the tissue section or in lumens) was identified by clustering the pixels of the H&E image by colour (k-means; k = 5). The lightest coloured cluster captured this empty space. Then the empty space pixels were grouped spatially by distance from each other, using the graph_from_adjacency_matrix function in igraph^15^ Connected groups of empty space pixels were then identified within this graph using the *components* function, implementing a connected components algorithm, with default parameters. This identified 110 lumen regions within the tissue. Lumen regions were classified by size to distinguish small sections of sinusoid (<150 pixels), small lumen (<600 pixels), and large lumen (>600 pixels). This translated to a mean diameter of 46 µm for small lumen and 129 µm for large. These lumen identified from the H&E image were overlaid with the 8 µm bin coordinates to assign bins to lumen. Proximity to lumen regions was defined as: within 16 µm (“adjacent”) and 80 µm (“proximal”). Cell composition was then compared between regions adjacent or proximal to either small or large lumen (Fig. 5a–d). Fibroblast and hepatic stellate cell abundance significantly varied by proximity to the lumen (adjacent vs. proximal; two-way ANOVA, p < 0.05), but was not significantly dependent on lumen size (large vs. small).

**Fig. 6:**
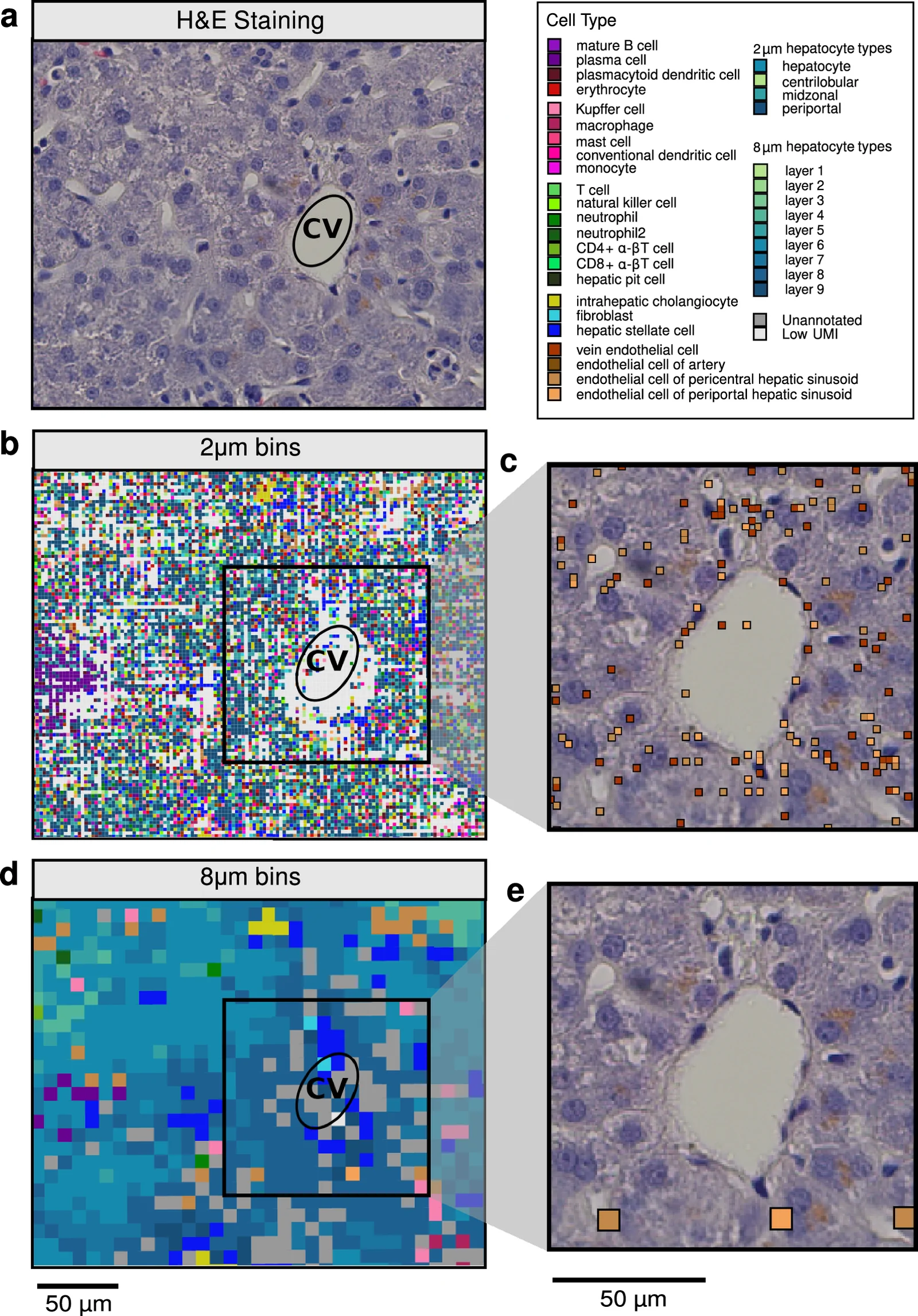
Though sparse in counts the 2 µm bin resolution can capture endothelial lining at central veins not captured at the 8 µm bin resolution. (**a**) H&E staining of a representative central vein (CV). (**b**) Cell type annotation at the 2 µm bin resolution. (**c**) Further zoom-in on the representative CV with only those 2 µm bins annotated as endothelial highlighted. Points are coloured by endothelial cell type to highlight the distribution of vein endothelial cells (*TAGLN* and *SPARCL1* high) versus endothelial cells of pericentral hepatic sinusoid (*CLEC4G* and *LYVE1* high). (**d**) Cell type annotation at the 8 µm bin resolution. (**e**) Further zoom-in on the representative CV with only those 8 µm bins annotated as endothelial highlighted.

Cell annotation was performed at the 8 µm bin resolution across the entire sample (Fig. 1b,c), which may limit the resolution of small cell types, as reflected by the number of 8 µm bins annotated as doublets (Fig. 2b). To capture smaller cell types, at one representative central vein region, RCTD was run on 2 µm bins with a cut off for low UMI of 20 (Fig. 6a). At this central vein, cells lining the vein were annotated by RCTD as endothelial cells and these cells were visually distinct in the H&E images (Fig. 6b,c). At this same central vein region, no 8 µm bins at the lining of the veins were annotated as endothelial cells (Fig. 6d,e). The ability to annotate the expected endothelial cells lining this vein demonstrates the utility of the 2 µm bin resolution to identify smaller cells that may be missed at 8 µm.

**Fig. 7:**
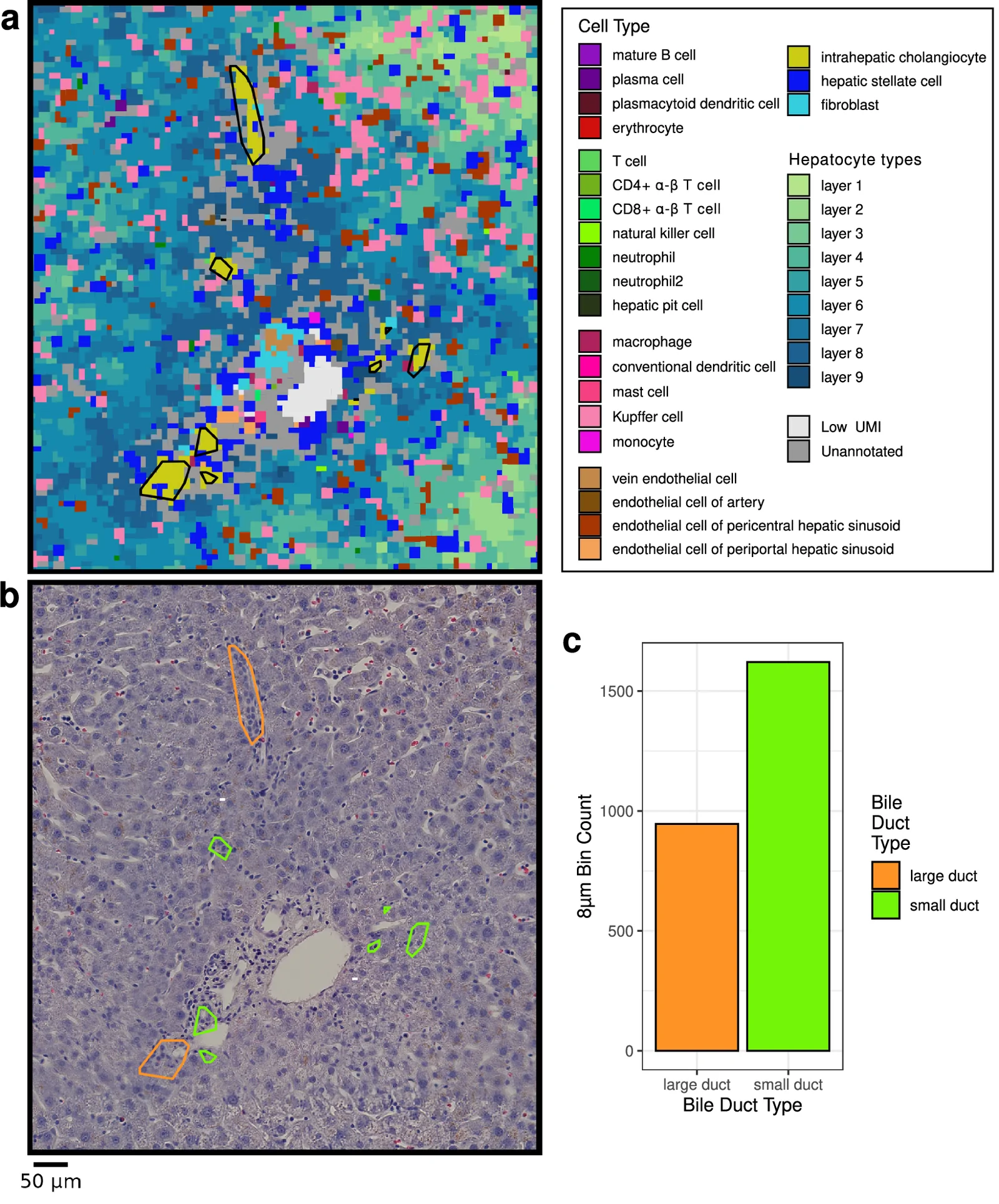
Bile ducts of different sizes can be identified. (**a**) Cell type annotation at the 8 µm bin resolution at representative periportal region. Transcriptionally identified bile ducts are highlighted with a black outlines. (**b**) H&E staining of a representative region with transcriptionally identified bile ducts are highlighted and coloured by size type. (**c**) Counts of cholangiocyte bins which were assigned to either small or large bile ducts.

As bile ducts are made up of adjacent cholangiocytes, we identified bile ducts by grouping neighboring bins annotated as intrahepatic cholangiocytes (Fig. 7a,b). A cell–cell adjacency matrix, based on Euclidean distances between cholangiocytes, was converted into an undirected graph using the *graph_from_adjacency_matrix* function in igraph^15^. As before, connected groups of cholangiocytes using the *components* function with default parameters. Of the 368 connected components of cholangiocytes, those containing more than 20 bins were classified as large bile ducts, those with 2–20 bins as small bile ducts, and groups with fewer than 2 bins were excluded as potential technical artifacts. This translated to a mean diameter of 26 µm for small ducts and 82 µm for large (Fig. 7c).

### Validation of gene expression measurements by immunohistochemistry

Immunohistochemical images were obtained from the Human Protein Atlas (https://www.proteinatlas.org) and the annotation of central and portal veins were confirmed by a liver pathologist (CT). The representative immunohistochemistry (IHC) stain shown is for: HAL - antibody HPA038547 patient id 2429; CYP1A2 - antibody CAB016531 patient id 3378.

## Data Record

Data are available^16^ NCBI Gene Expression Omnibus (GEO) under accession number GSE311383. The associated H&E images and annotation of the 8 µm bins^6^ are available at: https://doi.org/10.5281/zenodo.19609466.

## Technical Validation

**Fig. 8:**
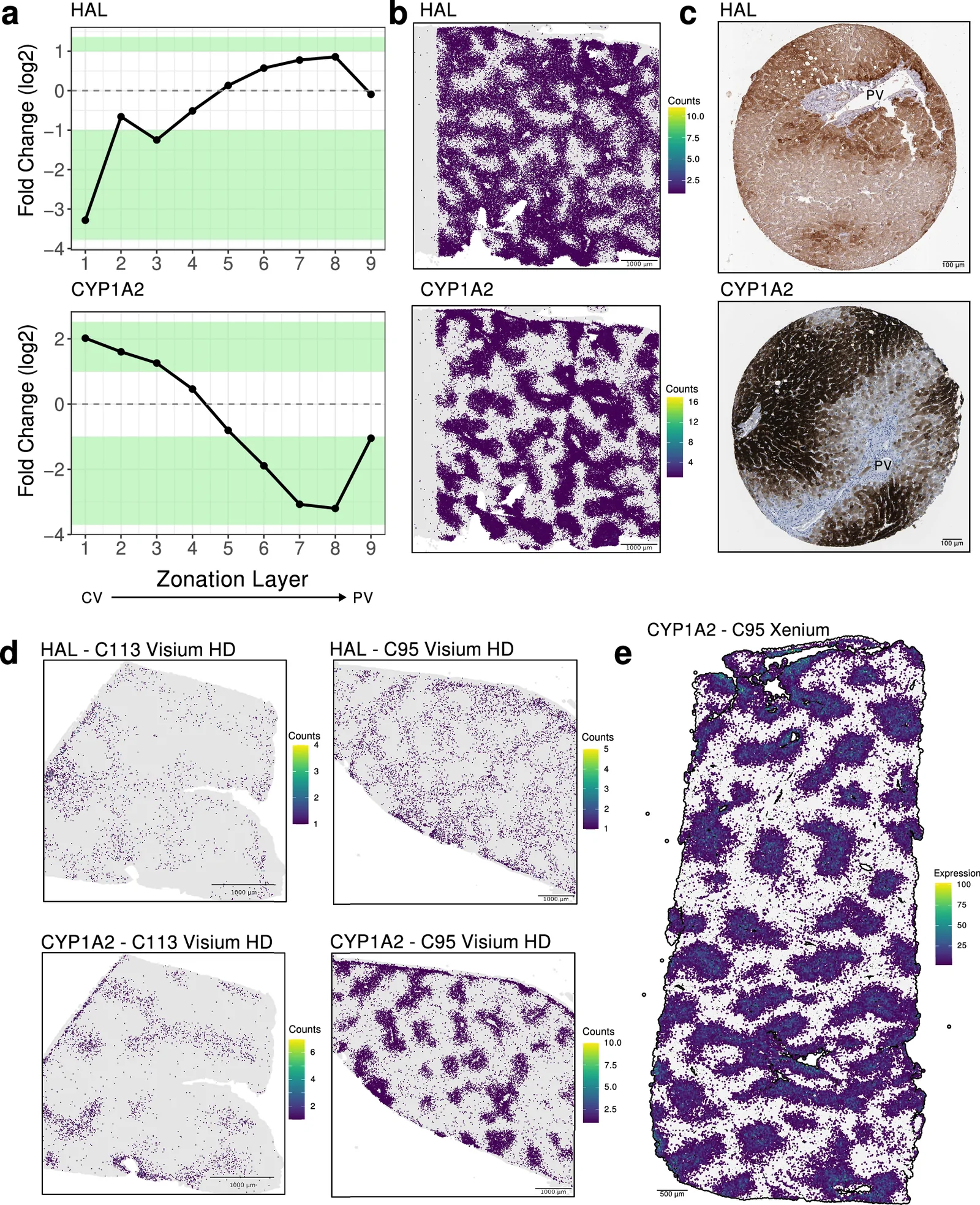
Markers of zonation can be identified in the Visium HD expression data and validated at the protein level. (**a**) Differential expression fold change of representative two zonated genes (*HAL* and *CYP1A2*). Fold change values compare all 8 µm Visium HD bins in a given zonation bin to all others for sample C107. (**b**) Expression of the two zonated genes across the entire C107 sample. (**c**) Representative IHC plots verifying zonation of these genes at the protein level. Annotations highlight portal veins (PV). (**d**) Expression of *HAL* and *CYP1A2* across the other two Visium HD samples (C95 and C113). (**e**) Expression of *CYP1A2* in the Xenium data available for C95.

To technically validate the data, we assessed its ability to capture a strong expected biological signal. In liver spatial transcriptomics, one of the strongest expected signals is hepatocyte zonation. We therefore wanted to validate the ability of the Visium HD to capture hepatocyte zonation. capture this pattern. Differential expression analysis of Visium HD bins revealed clear periportal and pericentral markers (Fig. 8a). Fold-change values were calculated by comparing all 8 µm bins assigned to a given zonation region against all other bins (Fig. 8a). The spatial distribution of these genes across the tissue section recapitulated the expected zonation patterns (Fig. 8b). To validate the ability of Visium HD to capture zonation, we examined both a well-established periportal zonation marker (*HAL*) and a pericentral zonation marker (*CYP1A2*). Importantly, protein-level validation using immunohistochemistry (IHC) data from the Human Protein Atlas confirmed the zonated expression of these markers (Fig. 8c), supporting the ability of Visium HD to capture physiologically relevant spatial organization in the liver. Beyond sample C107, the other two Visium HD samples (C95 and C113), although lower quality, still capture the zonation of these markers (Fig. 8d). For C95, previously published Xenium data are also available, and *CYP1A2* was included in the 477 gene panel^17^. This further validated the zonation signal captured by Visium HD (Fig. 8e).

## Usage Notes

An interactive Shiny app for exploring regions of interest are available at: faizanhasan20.github.io/VisiumHD-Liver.

For two samples additional spatial and dissociated single-cell data have been published^17^.

## Data Availability

All data supporting this study are publicly available. Spatial transcriptomics data are deposited in GEO under accession GSE311383. Associated H&E images are available at https://doi.org/10.5281/zenodo.19609466.

## Code availability

Reads were aligned and expression was quantified using Space Ranger (versions in Table 1). All analyses were performed in R (version 4.4.0). Bin annotation was carried out using Seurat (v5.3.0), spacexr/RCTD (v2.2.1), and BANKSY (v1.2.0). Graph-based analyses were performed using igraph (v2.1.4). Unless otherwise specified, analyses were run using default parameters. All code for analysis presented here is available at: github.com/faizanhasan20/VisiumHD-Liver.
